# Single tracer-based protocol for broad-spectrum kinase profiling in live cells with NanoBRET

**DOI:** 10.1016/j.xpro.2021.100822

**Published:** 2021-09-15

**Authors:** Matthew B. Robers, Jennifer M. Wilkinson, James D. Vasta, Lena M. Berger, Benedict-Tilman Berger, Stefan Knapp

**Affiliations:** 1 Promega Corporation, 2800 Woods Hollow Road, Madison, WI 53719, USA; 2 Structural Genomics Consortium, Goethe University Frankfurt, Buchmann Institute for Molecular Life Sciences, Max-von-Laue-Straße 15, 60438 Frankfurt am Main, Germany; Institute of Pharmaceutical Chemistry, Goethe University Frankfurt, Buchmann Institute for Molecular Life Sciences, Max-von-Laue-Straße 9, 60438 Frankfurt am Main, Germany

**Keywords:** cell biology, cell-based assays, high-throughput screening, molecular biology, molecular/chemical probes, biotechnology and bioengineering

## Abstract

This protocol is used to profile the engagement of kinase inhibitors across nearly 200 kinases in a live-cell context. This protocol utilizes one single kinase tracer (NanoBRET(TM) Tracer K10) that operates quantitatively at four different concentrations. Minimizing the number of tracers offers a significant workflow improvement over the previous protocol that utilized a combination of 6 tracers. Each NanoBRET(TM) kinase assay is built using commercially available plasmids and has been optimized for NanoLuc tagging orientation, diluent DNA, and tracer concentration.

For complete details on the use and execution of this protocol, please refer to Vasta et al. (2018).

## Highlights

• Target engagement is quantified against 192 full-length kinases in intact cells
• The method uses a single BRET tracer, at 4 different operating concentrations
• This method is simple and can be executed using common lab equipment
• Engagement selectivity differs in live cells vs cell free systems

## Graphical abstract

**Figure:**
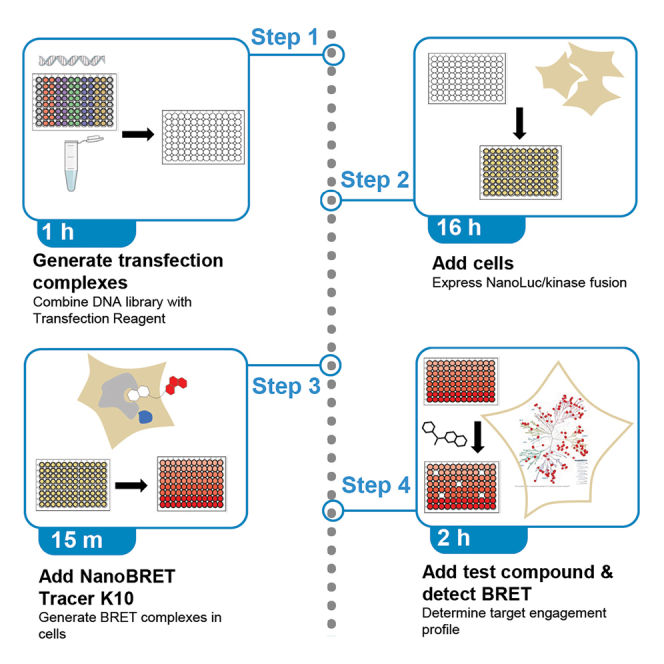

## Teaser

This protocol is used to profile the engagement of kinase inhibitors across nearly 200 kinases in a live-cell context. This protocol utilizes one single kinase tracer (NanoBRET Tracer K10) that operates quantitatively at four different concentrations. Minimizing the number of tracers offers a significant workflow improvement over the previous protocol that utilized a combination of 6 tracers. Each NanoBRET kinase assay is built using commercially available plasmids and has been optimized for NanoLuc tagging orientation, diluent DNA, and tracer concentration.

## Before you begin

### Prepare concentrated DNA stock solutions (0.2 mg/mL) and 2**×** working DNA solutions (20 μg/mL)

**Timing:1–10 h**

1. Table 1 describes the characteristics of each kinase and its performance in the NanoBRET(TM) assay with Tracer K10. Obtain this library of plasmids encoding NanoLuc/kinase fusions (from Promega individually or *en masse* as a collection).

**Table 1:** Summary of individual NanoBRET(TM) Kinase assay components and expected assay outcomes using control inhibitors CC1 or crizotinib

Kinase	Product # (promega)	Diluent DNA	Orientation of Nluc	[NanoBRET(TM) tracer K10], nM	BRET S:B (BRET_Tracer_ / BRET_no tracer_)	CC1 % occupancy, 300 nM	St dev	Crizotinib % occupancy, 1 uM	St dev
LRRK2	NV340A	Transfection Carrier	C	25	3.7	98	2.0	−9	15.2
MAPK6	NV169A	Transfection Carrier	N	25	7.4	99	0.8	2	2.4
IRAK3	NV144A	Transfection Carrier	N	25	4.5	98	0.8	42	4.4
TEK	NV215A	Transfection Carrier	C	25	3.7	90	1.0	37	4.7
TNK1	NV218A	Transfection Carrier	N	25	10.0	94	0.8	−6	4.1
GAK	NV142A	Transfection Carrier	N	25	14.9	97	0.5	−14	1.5
MAPK4	NV168A	Transfection Carrier	N	25	26.8	97	1.2	−12	3.4
AAK1	NV100A	Transfection Carrier	N	25	12.2	99	0.3	−14	0.9
AURKA	NV104A	Transfection Carrier	C	25	6.8	93	1.0	30	7.7
AURKC	NV106A	Transfection Carrier	C	25	10.6	92	1.8	48	4.2
AURKB	NV105A	Transfection Carrier	C	25	10.7	94	1.6	46	5.9
NUAK1	NV183A	Transfection Carrier	N	25	3.4	98	2.4	36	6.6
LATS2	NV151A	Transfection Carrier	C	25	5.0	79	7.1	−10	6.2
RPS6KA3	NV200A	Transfection Carrier	N	25	3.6	97	0.9	−7	7.7
SNF1LK2	NV206A	Transfection Carrier	N	25	16.9	99	0.5	0	3.4
MYLK2	NV177A	Transfection Carrier	C	25	6.2	99	0.4	−16	8.3
AXL	NV107A	Transfection Carrier	C	250	2.9	63	3.8	68	1.5
FGFR3	NV136A	Transfection Carrier	C	250	3.4	84	4.1	14	6.8
FLT3	NV139A	Transfection Carrier	C	250	3.4	79	3.7	−4	8.6
IGF1R	NV326A	Transfection Carrier	C	250	2.1	78	3.8	18	8.2
INSR	NV327A	Transfection Carrier	C	250	2.9	83	2.5	20	2.1
LIMK2	NV153A	Transfection Carrier	C	250	4.2	76	3.6	32	9.8
TEC	NV214A	Transfection Carrier	N	250	4.6	78	1.4	−7	6.7
TIE1	NV217A	Transfection Carrier	C	250	9.9	67	7.2	35	9.9
CLK1	NV113A	Transfection Carrier	N	250	7.3	98	0.7	0	3.4
SBK3	NV421A	Transfection Carrier	N	250	4.1	90	4.4	41	5.6
NEK9	NV180A	Transfection Carrier	N	250	7.9	65	5.2	1	5.4
NEK3	NV179A	Transfection Carrier	N	250	16.1	44	9.6	−2	14.5
NIM1K	NV381A	Transfection Carrier	C	250	4.6	82	4.9	−3	1.7
STK36	NV433A	Transfection Carrier	N	250	7.1	89	3.8	−6	6.5
ULK2	NV222A	Transfection Carrier	N	250	10.5	78	8.0	−1	1.9
ULK3	NV449A	Transfection Carrier	N	250	23.1	72	5.2	2	6.5
BRSK1	NV249A	Transfection Carrier	N	250	8.3	78	7.1	−1	4.0
MAP3K10	NV156A	Transfection Carrier	N	250	4.5	54	3.3	−1	4.4
MAP3K9	NV160A	Transfection Carrier	N	250	2.9	60	4.3	−1	6.2
MYLK3	NV374A	Transfection Carrier	C	250	8.5	72	9.1	−8	6.4
PHKG1	NV186A	Transfection Carrier	N	250	5.9	86	3.1	−3	6.3
STK33	NV211A	Transfection Carrier	N	250	3.1	90	3.4	−3	3.9
STK4	NV435A	Transfection Carrier	N	250	4.8	67	2.4	11	7.6
TLK1	NV443A	Transfection Carrier	C	250	2.8	5	1.9	−5	3.8
FGFR1	NV134A	Transfection Carrier	C	100	3.3	95	1.8	0	3.8
FGFR2	NV135A	Transfection Carrier	C	100	3.5	94	0.7	10	1.8
MUSK	NV176A	Transfection Carrier	C	100	3.3	89	4.3	80	2.8
NTRK1	NV181A	Transfection Carrier	C	100	4.1	68	4.5	71	2.5
RET	NV195A	Transfection Carrier	C	100	6.0	90	1.5	4	4.5
NTRK2	NV182A	Transfection Carrier	C	100	4.6	80	3.4	85	2.3
TNK2	NV445A	Transfection Carrier	C	100	6.8	73	4.0	13	6.7
LTK	NV154A	Transfection Carrier	C	100	4.2	71	4.3	87	0.9
BRAF(V600E)	NV248A	Transfection Carrier	C	100	3.9	39	5.8	−4	3.9
IRAK4	NV145A	Transfection Carrier	C	100	6.5	98	1.4	6	7.0
ITK	NV146A	Transfection Carrier	N	100	3.4	92	1.8	−1	6.2
JAK2 (V617F)	NV330A	Transfection Carrier	C	100	3.3	90	2.8	65	8.3
MAP3K11	NV157A	Transfection Carrier	N	100	4.6	71	4.9	0	6.0
PTK2	NV192A	Transfection Carrier	N	100	3.3	88	1.2	40	4.0
PTK6	NV194A	Transfection Carrier	C	100	3.7	100	0.6	−2	1.2
PTK2B	NV193A	Transfection Carrier	C	100	2.2	91	2.2	47	5.1
BMP2K	NV109A	Transfection Carrier	N	100	8.9	94	1.6	11	6.0
NEK5	NV379A	Transfection Carrier	N	100	16.2	85	1.2	−6	2.1
STK16	NV209A	Transfection Carrier	N	100	13.4	85	1.9	−13	6.2
TBK1	NV213A	Transfection Carrier	N	100	7.5	78	4.2	−7	10.8
ULK1	NV221A	Transfection Carrier	N	100	14.2	94	1.9	−1	5.5
MAP4K2	NV162A	Transfection Carrier	N	100	4.5	76	2.0	77	10.7
WEE1	NV223A	Transfection Carrier	C	100	5.3	91	1.0	−9	2.5
MYLK4	NV375A	Transfection Carrier	C	100	6.1	99	0.7	−8	8.4
MARK4	NV173A	Transfection Carrier	N	100	4.4	90	2.0	−15	4.5
PRKAA1	NV410A	Transfection Carrier	N	100	11.3	92	1.5	−1	4.9
PRKAA2	NV189A	Transfection Carrier	N	100	12.6	97	0.3	−6	2.2
RPS6KA1	NV198A	Transfection Carrier	N	100	7.9	89	1.9	−6	2.5
RPS6KA2	NV199A	Transfection Carrier	N	100	4.7	79	4.2	−7	7.5
RPS6KA4	NV201A	Transfection Carrier	N	100	6.6	85	0.7	−11	6.0
RPS6KA6	NV202A	Transfection Carrier	N	100	3.0	89	2.3	−10	7.3
SIK1	NV203A	Transfection Carrier	N	100	10.2	93	1.6	−4	4.7
CLK4	NV115A	Transfection Carrier	C	100	2.5	101	1.4	−19	4.6
MAPK8	NV170A	Transfection Carrier	N	100	5.8	70	7.8	−16	4.2
MAPK9	NV171A	Transfection Carrier	N	100	9.4	70	6.4	−11	2.1
IKBKE	NV143A	Transfection Carrier	N	100	7.7	74	5.2	21	2.8
LATS1	NV150A	Transfection Carrier	C	100	5.1	68	5.7	−10	3.9
PRKX	NV191A	Transfection Carrier	C	100	3.6	65	4.9	−14	7.9
CSNK2A2	NV119A	Transfection Carrier	C	100	5.5	87	3.0	−14	5.8
HIPK4	NV324A	Transfection Carrier	N	100	7.8	97	0.4	−9	6.1
STK10	NV426A	Transfection Carrier	C	1000	2.8	80	3.4	35	3.0
FGFR4	NV137A	Transfection Carrier	C	1000	3.5	68	4.0	3	1.6
MAP4K1	NV161A	Transfection Carrier	N	1000	3.9	70	4.5	14	4.1
MERTK	NV356A	Transfection Carrier	C	1000	2.2	48	3.9	58	3.3
MET	NV175A	Transfection Carrier	C	1000	5.1	42	8.5	95	1.1
RON	NV418A	Transfection Carrier	C	1000	3.9	32	9.9	86	4.8
TYRO3	NV448A	Transfection Carrier	C	1000	2.5	47	0.5	49	2.2
LCK	NV152A	Transfection Carrier	C	1000	3.8	78	4.2	36	5.7
LIMK1	NV339A	Transfection Carrier	C	1000	5.2	69	5.6	29	5.5
EPHA1	NV122A	Transfection Carrier	C	1000	3.3	74	3.2	75	2.2
EPHA4	NV124A	Transfection Carrier	C	1000	2.8	33	5.2	53	4.4
EPHA6	NV126A	Transfection Carrier	C	1000	3.9	38	1.7	64	5.1
EPHA7	NV127A	Transfection Carrier	C	1000	4.4	51	4.6	38	2.4
EPHB1	NV307A	Transfection Carrier	C	1000	3.4	65	2.4	68	2.0
EPHB4	NV131A	Transfection Carrier	C	1000	3.1	53	4.9	62	3.6
FYN	NV141A	Transfection Carrier	C	1000	3.9	65	7.5	−2	12.3
ABL2	NV233A	Transfection Carrier	N	1000	2.9	72	5.5	43	3.9
BMX	NV110A	Transfection Carrier	C	1000	4.5	60	6.3	7	4.3
BTK	N244A	Transfection Carrier	C	1000	6.8	75	4.4	1	1.3
FER	NV133A	Transfection Carrier	C	1000	2.8	66	4.9	16	7.9
FES	NV309A	Transfection Carrier	C	1000	3.0	43	7.6	1	2.5
JAK3	NV147A	Transfection Carrier	C	1000	4.7	45	4.8	2	4.4
SRMS	NV425A	Transfection Carrier	N	1000	2.7	18	4.3	0	4.9
TXK	NV220A	Transfection Carrier	C	1000	2.6	60	13.1	18	2.8
CLK2	NV114A	Transfection Carrier	C	1000	4.1	89	2.2	1	4.7
DYRK1A	NV303A	Transfection Carrier	N	1000	2.6	78	4.4	0	5.3
DYRK1B	NV121A	Transfection Carrier	N	1000	3.3	87	3.6	0	2.1
ERN1	NV132A	Transfection Carrier	C	1000	3.6	83	1.3	−3	8.1
ERN2	NV308A	Transfection Carrier	C	1000	5.3	61	7.4	−9	13.9
HIPK2	NV322A	Transfection Carrier	N	1000	3.3	82	2.8	5	4.4
HIPK3	NV323A	Transfection Carrier	N	1000	1.9	70	14.3	−4	14.6
ICK	NV325A	Transfection Carrier	N	1000	2.6	47	11.0	3	15.4
CDK1	NV270A	CycB1 (NV260A)	C	1000	3.0	71	7.1	7	2.9
CDK2	NV278A	CycE1 (NV264A)	C	1000	12.5	80	4.0	3	1.6
CDK3	NV280A	CycE1 (NV264A)	C	1000	6.9	85	3.5	6	1.3
CDK4	NV281A	CycD3 (NV263A)	N	1000	8.2	93	2.2	4	4.2
CDK5	NV112A	CDK5R1 (NV282A	C	1000	13.3	85	3.6	1	0.8
CDK6	NV284A	CycD1 (NV262A)	N	1000	4.1	89	2.2	0	3.0
CDK7	NV285A	Transfection Carrier	N	1000	3.3	83	4.1	32	1.3
CDK9	NV287A	CycK (NV266A)	N	1000	3.8	68	4.1	4	5.5
CDK10	NV271A	CycL2 (NV267A)	C	1000	2.9	50	3.7	8	0.5
CDK14	NV272A	CycY (NV269A)	C	1000	4.3	82	1.3	0	2.4
CDK15	NV273A	CycY (NV269A)	N	1000	3.5	78	2.1	3	2.8
CDK16	NV274A	CycY (NV269A)	C	1000	7.2	96	0.6	2	0.4
CDK17	NV275A	CycY (NV269A)	C	1000	9.6	95	0.8	3	3.6
CDK18	NV276A	CycY (NV269A)	C	1000	11.4	86	1.4	2	2.1
CDKL2	NV289A	Transfection Carrier	N	1000	6.7	95	1.3	21	0.7
CDK20	NV279A	CycH (NV265A)	N	1000	4.3	54	4.9	−11	5.0
CDKL1	NV288A	Transfection Carrier	N	1000	5.1	64	3.2	2	0.9
CSNK2A1	NV298A	Transfection Carrier	C	1000	2.8	64	2.6	5	6.7
CDKL3	NV290A	Transfection Carrier	N	1000	2.2	83	4.5	8	2.0
CDKL5	NV291A	Transfection Carrier	N	1000	4.6	84	1.3	4	1.8
JNK3	NV148A	Transfection Carrier	C	1000	3.1	36	3.9	−4	0.8
MAPK11	NV165A	Transfection Carrier	N	1000	5.4	35	2.3	0	2.6
MAPK14	NV166A	Transfection Carrier	C	1000	3.7	22	1.5	0	1.7
NLK	NV382A	Transfection Carrier	C	1000	3.2	30	3.4	−1	3.5
NEK11	NV377A	Transfection Carrier	N	1000	3.9	52	4.2	7	3.6
NEK1	NV376A	Transfection Carrier	N	1000	5.9	38	1.7	3	4.1
NEK2	NV178A	Transfection Carrier	N	1000	3.4	46	2.7	6	6.1
NEK4	NV378A	Transfection Carrier	N	1000	3.8	25	0.8	−12	5.6
PAK4	NV184A	Transfection Carrier	C	1000	11.1	25	5.5	1	2.4
MAP4K3	NV163A	Transfection Carrier	N	1000	7.6	50	1.4	8	1.5
STK11	NV208A	Transfection Carrier	N	1000	3.5	81	2.3	−1	0.9
SLK	NV205A	Transfection Carrier	N	1000	2.5	87	3.8	36	2.9
DAPK2	NV299A	Transfection Carrier	N	1000	2.5	53	3.4	3	1.6
MAP3K2	NV347A	Transfection Carrier	C	1000	3.2	47	2.9	6	1.7
PLK2	NV408A	Transfection Carrier	N	1000	3.0	71	3.2	4	8.5
PLK3	NV409A	Transfection Carrier	N	1000	3.2	52	0.8	4	1.9
PLK4	NV188A	Transfection Carrier	N	1000	8.6	52	3.9	4	1.2
STK35	NV432A	Transfection Carrier	C	1000	3.4	60	5.9	16	3.3
STK17B	NV427A	Transfection Carrier	C	1000	1.9	56	2.5	0	3.8
TLK2	NV444A	Transfection Carrier	C	1000	3.7	1	3.9	0	3.2
BRSK2	NV111A	Transfection Carrier	N	1000	15.9	79	2.5	3	2.4
MARK2	NV172A	Transfection Carrier	N	1000	4.2	76	1.7	4	2.1
MELK	NV174A	Transfection Carrier	N	1000	5.8	43	6.8	−2	2.4
CSNK1A1L	NV295A	Transfection Carrier	N	1000	3.6	19	4.3	−6	2.9
CSNK1D	NV296A	Transfection Carrier	N	1000	3.5	30	2.3	−4	1.4
CSNK1G2	NV118A	Transfection Carrier	N	1000	3.4	47	1.2	1	2.8
SIK3	NV204A	Transfection Carrier	N	1000	12.8	62	0.8	0	3.3
SNRK	NV424A	Transfection Carrier	N	1000	4.0	68	3.7	−4	7.7
CAMK1	NV253A	Transfection Carrier	N	1000	2.4	54	2.5	7	2.6
CAMK2A	NV256A	Transfection Carrier	N	1000	5.8	64	0.7	3	2.3
CAMK2D	NV257A	Transfection Carrier	N	1000	5.7	68	1.3	3	2.2
CHEK2	NV293A	Transfection Carrier	C	1000	2.4	40	4.4	0	5.0
DCLK3	NV300A	Transfection Carrier	C	1000	2.4	61	2.7	0	3.4
MKNK2	NV371A	Transfection Carrier	N	1000	2.6	64	3.0	1	4.6
PHKG2	NV388A	Transfection Carrier	N	1000	2.7	50	1.4	3	2.5
MAP3K3	NV349A	Transfection Carrier	C	1000	3.1	36	3.3	4	1.9
RIOK2	NV196A	Transfection Carrier	N	1000	7.1	31	3.4	9	6.8
MAP4K5	NV350A	Transfection Carrier	C	1000	3.2	26	1.2	36	4.0
MAST3	NV353A	Transfection Carrier	N	1000	2.5	44	8.8	−1	6.6
MAST4	NV354A	Transfection Carrier	N	1000	2.2	46	6.0	6	6.4
STK32B	NV210A	Transfection Carrier	N	1000	3.8	18	4.3	−1	1.9
STK3	NV430A	Transfection Carrier	N	1000	3.9	43	3.5	1	8.8
STK38	NV212A	Transfection Carrier	C	1000	6.8	24	0.5	1	2.2
STK38L	NV434A	Transfection Carrier	C	1000	3.9	20	2.1	5	2.8
PAK6	NV386A	Transfection Carrier	C	1000	3.3	24	13.4	−7	23.7
AKT2	NV103A	Transfection Carrier	C	1000	3.5	22	1.7	−1	4.7
PKMYT1	NV187A	Transfection Carrier	C	1000	2.6	30	1.1	6	1.7
PRKACA	NV190A	Transfection Carrier	C	1000	6.9	56	3.1	−1	1.4
PRKACB	NV411A	Transfection Carrier	C	1000	3.6	26	3.5	5	3.5
PRKCE	NV412A	Transfection Carrier	C	1000	3.3	30	8.1	−6	6.1
SGK1	NV422A	Transfection Carrier	C	1000	3.0	43	4.5	−1	1.1
WEE2	NV450A	Transfection Carrier	N	1000	2.8	69	2.7	−4	2.6
RIPK1	NV417A	Transfection Carrier	N	1000	2.8	25	1.8	23	3.9
RIPK2	NV197A	Transfection Carrier	N	1000	9.1	64	1.5	43	0.5
TNNI3K	NV446A	Transfection Carrier	C	1000	4.0	65	0.9	85	1.4
MLTK	NV372A	Transfection Carrier	N	1000	4.4	62	5.4	−3	8.7
MAP3K12	NV158A	Transfection Carrier	N	1000	3.7	39	1.0	12	6.8
MAP3K19	NV346A	Transfection Carrier	C	1000	3.0	8	4.4	6	5.7
MAP3K21	NV348A	Transfection Carrier	N	1000	3.2	33	0.6	3	5.3
MAP3K4	NV159A	Transfection Carrier	C	1000	4.8	90	1.7	−1	2.6

***Note:*** Each plasmid has been purified with low endotoxin levels. If this library is being generated de novo, be sure to utilize purification methods that yield low endotoxin.

2. **Prepare concentrated DNA stock solutions for long-term storage.** Prepare each DNA in this library in a diluent DNA (either Transfection Carrier DNA or cyclin DNA as described in Table 1) to generate 0.18 mg/mL diluent DNA and 0.02 mg/mL kinase/Nluc fusion DNA. The total DNA concentration of each solution should be 0.2 mg/mL

***Note:*** This material at 0.2 mg/mL can be stored long term (at least 6 months) when frozen at – 20°C. For long-term storage, seal each plate with foil tape covers at - 20°C. Ideally, plates stored long-term will be heat-sealed. To further minimize evaporation during storage, enclose each plate in an air-tight plastic bag. When thawing the DNA plate, spin the DNA plates prior to removing the foil cover.

3. **From the concentrated stock solutions, prepare 2× working DNA solutions for day-to-day experimentation.** Dilute the stock 0.2 mg/mL plasmid DNA solutions 1:10 into nuclease-free TE buffer to achieve a total final concentration of 20 μg/mL DNA in TE buffer.
4. As described above, seal each plate with foil tape covers to avoid evaporation. To further minimize evaporation during storage, enclose each plate in an air-tight plastic bag. Ideally, plates stored long-term will be heat-sealed.
5. Store these 20 μg/mL solutions for up to 4 weeks at −80°C and avoid greater than 5**×** freeze/thaw cycles. When thawing the DNA plate for transfection, spin the DNA plates prior to removing the foil cover. This will minimize the cross-contamination. Reseal with fresh seals after each use.

## Key resources table

**Table:** 

REAGENT or RESOURCE	SOURCE	IDENTIFIER
**Experimental models: cell lines**		

HEK293 cells	ATCC	CRL-1573

**Chemicals, peptides, and recombinant proteins**		

OptiMEM	Gibco	11058–021
DMEM	Gibco	12430–054
Fetal Bovine Serum (FBS)	VWR	89510–194
FuGENE HD	Promega	E2312
Trypsin	Gibco	25300–056
DMSO	Sigma	D2650-5X10ML
CC1 pan-Kinase Inhibitor	Promega	N2661
Crizotinib	Selleckchem	S1068
TE Buffer, Sterile, Nuclease-free	Promega	V6231

**Critical commercial assays**		

NanoBRET(TM) Tracer Dilution Buffer	Promega	N219B
NanoBRET-TE Complete Tracer K10 Kit	Promega	N2641

**Recombinant DNA**		

Kinase/NanoLuc Plasmid Library for Live Cell Profiling with NanoBRET(TM) Tracer K10. (see Table 1 )	Promega	https://www.promega.com/resources/guides/kinase-target-engagement-assay-selection-table/#sort=%40kinasez32xname%20ascending
NanoLuc(R) Control Vector	Promega	N1091
Transfection Control Vector (MET-Nluc)	Promega	NV175A
Transfection Carrier DNA	Promega	E4882

**Other**		

Sterile, white, opaque, TC-treated 96 well plates (assay plates for cell seeding)	Corning	3917
96-well polypropylene plate (for transfection complex preparation)	Thermo Scientific Nunc	12–565-438
96-well plates (for DNA storage)	Axygen	P-96-450V-C
Axygen Aluminum sealing film	Axygen	PCR-AS-600
NanoBRET™-compatible luminometer	n/a	n/a

## Step-by-step method details

### Day 1: Cell transfection

**Timing:1 h**

This step is designed to deliver each plasmid DNA into HEK293 cells to generate individual cell populations expressing selected kinase/DNA fusion proteins.

1. Passage HEK-293 cells with DMEM + 10% FBS (growth medium) one day prior to the transfection (2 days prior to assay). Avoid use of cells that have exceeded 50 passages.
2. Ensure that cells are 70–90% confluent on the day of transfection
3. Trypsinize the cells and inactivate the trypsin using complete growth medium. Centrifuge the cells at 200 **×** *g* / 4°C to generate a cell pellet.
4. Resuspend HEK293 cells for transfection and adjust the cell density to 2 **×** 10^5^/mL in Opti-MEM + 1% FBS.
5. Add 100 μL/well of HEK293 cells to each well of a TC-treated, 96-well plate.

6. Prepare Transfection complexes.
  a. Estimate the required volume of transfection complex, determine the number of data points needed for each kinase.
    The following controls or conditions are recommended:
    **CRITICAL:**Zero BRET Control: NanoLuc® control vector (lacking a kinase fusion) must be used for each experiment to define 100% Fractional Occupancy.
    ***Note:*** NanoLuc® control vector must be diluted in Transfection Carrier DNA prior to use. Dilute 1 part NanoLuc® control vector to 9 parts Transfection Carrier DNA. This DNA solution should then be diluted with TE Buffer to the same final working concentration (20μg/mL) as the kinase plasmids prior to creating transfection complexes.
    ***Note:*** Include this control on each assay plate. If desired, Tracer can be added to these wells to prove that there is no nonspecific BRET observed from the tracer.
    **CRITICAL:**100% BRET Control: This is Tracer + DMSO but without test compound. Use this control for each kinase to define 0% Fractional Occupancy.
    i. Technical replicates (recommended) can be used for each control and test compound sample
    ii. **Transfection Control** (recommended): Use this control to determine if the transfection was successful prior to executing the complete NanoBRET assay. Process this sample prior to the assay setup on day 2**. Prepare this transfection on a separate plate.** Transfect at least 4 wells for this purpose. The MET-NanoLuc plasmid is an ideal DNA for this purpose. Dilute the MET-NanoLuc control plasmid into Carrier DNA to match the conditions used for the experimental sample.
  b. Assemble 2**×** Kinase DNA solutions and transfer to a sterile 96-well polypropylene plate:
    ***Note:*** For each well of analysis, you will require 3 μL of each 2**×** Kinase DNA solution at 20μg/mL.
    ***Note:*** If frozen, we recommend thawing the DNA quickly at 37°C and centrifuging the plate or strip briefly to ensure the solution is at the bottom of the vessel.
  c. Prepare 2**×** Fugene HD solution:
    i. Dilute Fugene HD to a final concentration of 60μL/mL in room temperature (22°C–26°C) OptiMEM in a sterile conical tube, directly into the liquid.
      ***Note:*** For each well of analysis, prepare 3 μL of 2**×** Fugene HD solution.
  d. Add equal parts of the 2**×** Fugene HD solution to the 2**×** Kinase DNA solutions.
  e. Mix on an orbital shaker for 15 s at 400 rpm.
  f. Allow complexes to form for 30 min at room temperature.
    ***Note:*** For experienced users, the cells can be prepared during this step.

7. Transfer 5 μL per well of the Transfection Complex into 100 μL/well of the 96-well plate with the HEK293 cells.
8. Incubate 16–24 h at 37°C and 5% CO_2_.

***Note:*** Allow a minimum of 16 h for transfection to occur, ideally between 20–24 h.

### Day 2: Verify transfection efficiency with the transfection control samples

**Timing:30 min**

9. Prepare 3**×** Complete NanoBRET^TM^ Nano-Glo® Substrate (included in the NanoBRET(TM) K10 Complete Kit) in OptiMEM without serum or phenol red. This solution consists of a 1:166 dilution of NanoBRET^TM^ Nano-Glo® Substrate plus a 1:500 dilution of Extracellular NanoLuc Inhibitor in OptiMEM without serum or phenol red. Mix gently by inverting 5–10 times in a conical tube. (The final concentration of Extracellular NanoLuc inhibitor in the 3**×** solution is 60 μM, for a working concentration of 20 μM.)

***Note:*** 3**×** solutions should be used within 1.5 h of preparation.

10. To wells with transfected cells: Add 50μL per well of 3**×** Complete NanoBRET^TM^ Nano-Glo® Substrate with NanoBRET^TM^ Extracellular Inhibitor for a 96-well plate. Incubate 2–3 min at room temperature (RT).
11. To the empty wells that are on the opposite side of the plate: Add 50μL per well of 3**×** Complete NanoBRET^TM^ Nano-Glo® Substrate with NanoBRET^TM^ Extracellular Inhibitor for a 96-well plate. Incubate 2–3 min at RT. Note that measuring background wells adjacent to sample wells may result in signal bleed through. Therefore, to accurately quantify background luminescence, use wells of the plate that are not adjacent to wells that contain NanoLuc expression..
12. Following addition of NanoBRET^TM^ Nano-Glo® Substrate, measure donor emission (e.g., 450 nm) and acceptor emission (e.g., 610 nm or 630 nm) using a NanoBRET™-compatible luminometer.
13. Determine the signal-to-background (S/B) in the 450 nM channel.
  a. Calculate S/B.
    i. 450 nm relative light units (RLUs) (transfected cells + NanoGlo reagents) / RLUs (NanoGlo reagents alone)
  b. The S/B should be >1000 to support proceeding with a large scale assay. Ideally, S/B is 1 **×** 10^4^–1 **×** 10^5^.

### Cellular treatment of compounds and NanoBRET(TM) tracer

**Timing: 2–3 h**

This step is required for the cellular system to achieve equilibrium. This involves introduction of NanoBRET(TM) Tracer K10 at concentrations appropriate to quantify occupancy of each kinase. NanoBRET(TM) tracers generally equilibrate after short incubation times, however 2 h is recommended as a general incubation time for most kinase inhibitors. Longer incubations may be required for slow-binding inhibitors.

#### Addition of NanoBRET tracers

14. Based on the plate layout, calculate the number of data points needed for each concentration bin.
  a. Refer to Table 1 for a list of the four tracer conditions recommended (25 nM, 100 nM, 250 nM, 1000 nM). These concentrations are recommended based on kinase affinity measurements.

**CRITICAL:** For quantitative target engagement analysis, use NanoBRET(TM) tracers at or below their apparent affinity for each kinase. Therefore, tracer concentration should not exceed the values provided in Table 1. Although increasing tracer concentration will often produce a stronger BRET signal, the occupancy results may deviate due to over-saturation of the kinase target with tracer.

15. Prepare Complete 20**×** NanoBRET(TM) Tracer Reagent.
  a. First, prepare the 100**×** solution of the NanoBRET(TM) Tracer in DMSO.
    i. 1 μM concentration bin: Prepare 100 μM tracer (1:4 dilution of master stock)
    ii. 250 nM concentration bin: Prepare 25 μM tracer (1:16 dilution of master stock) into DMSO)
    iii. 100 nM concentration bin: Prepare 10 μM tracer (1:40 dilution of master stock into DMSO)
    iv. 25 nM concentration bin: Prepare 2.5 μM tracer (1:160 dilution of master stock into DMSO)
  b. From the 100**×** Tracer solutions, add tracer dilution buffer to generate 20**×** Complete Tracer Dilution Buffer
    i. Dilute 1 part 100**×** tracer with 4 parts tracer dilution buffer

***Note:*** To prepare the Complete 20**×** NanoBRET^TM^ Tracer Reagent, add the concentrated tracer stock and DMSO into a conical tube first, then mix. Then, to the resultant solution, add the Tracer Dilution Buffer and mix. For dispensing, add 20**×** tracer into a polypropylene (not polystyrene) trough.

16. Add 5μL of Complete 20**×** NanoBRET(TM) Tracer Reagent (+ tracer) per well to transfected cells, directly into the liquid.
17. Mix on an orbital shaker for 15 s at 900 RPM at room temperature.

#### Addition of test compounds

18. Prepare 10**×** test compounds in OptiMEM
  a. Dilute the test compound to 1000**×** in DMSO (or the test compound solvent). Example fractional occupancy profiling data is shown below for crizotinib (1 uM final concentration) or CC1 (300 nM concentration).
  b. Dilute the 1000**×** test compound to 10**×** in Opti-MEM.

***Note:*** For the DMSO control (100% BRET Control), dilute 10μL DMSO to 1mL with OptiMEM

19. Add 10μL per well of 10**×** test compound or 10μL of the DMSO control to the 96-well plates containing cells with 1**×** tracer. Mix on orbital shaker for 15 s at 900 rpm.
20. Incubate the plate at 37°C + 5% CO_2_ incubator for 2hr. Allow plate to cool to RT for ∼15 min, then proceed to NanoBRET^TM^ Assay section below.
21. Immediately before the BRET measurements, prepare 3**×** Complete NanoBRET^TM^ Nano-Glo® Substrate in OptiMEM without serum or phenol red. This solution consists of a 1:166 dilution of NanoBRET^TM^ Nano-Glo® Substrate plus a 1:500 dilution of Extracellular NanoLuc Inhibitor in OptiMEM without serum or phenol red. Mix gently by inverting 5–10 times in a conical tube. (The final concentration of Extracellular NanoLuc inhibitor in the 3**×** solution is 60 μM, for a working concentration of 20 μM.)
  a. Note: 3**×** solutions should be used within 1.5 h after preparation.
22. Add 50μL per well of 3**×** Complete NanoBRET^TM^ Nano-Glo® Substrate with NanoBRET^TM^ Extracellular Inhibitor for a 96-well plate. Incubate 2–3 min at RT. Following addition of NanoBRET^TM^ Nano-Glo® Substrate, measure donor emission (e.g., 450 nm) and acceptor emission (e.g., 610 nm or 630 nm) using a NanoBRET™-compatible luminometer.

## Quantification and statistical analysis

BRET Quantitation

1. To generate raw BRET ratio values, divide the acceptor emission value (e.g., 610 nm) by the donor emission value (e.g., 450 nm) for each sample.

Fractional occupancy Quantitation

2. Determine Fractional Occupancy with the following equation;
  ***% Occupancy = [1 – (X – Z)/( Y – Z)]∗100***
  Where:
    X = BRET in the presence of the test compound and tracer,
    Y = BRET in the presence of the 100% BRET Control (Tracer + DMSO)
    Z = BRET for the Zero BRET Control, e.g.,
      a. BRET from the NanoLuc Control Vector
      b. BRET from Kinase/NanoLuc fusion + saturating dose of control test compound.

Generate kinase target engagement dendrogram illustrations

3. Navigate to http://www.kinhub.org/kinmap/
4. Click on the “text” tab.
5. Enter in the kinase names (either in columns or linearly separated by commas) you would like represented on the dendrogram using the required syntax.

Note, entering simple kinase names (protein or gene) will be represented in the default annotation (small red circle). Follow the instructions in the “help” subtab, considering the example code provided in the “examples” subtab to use directives to change the symbol annotation if desired.

6. Click the “plot” button to annotate the dendrogram.
7. Download your dendrogram image by clicking the “download” button.

## Expected outcomes

**Figure 1:**
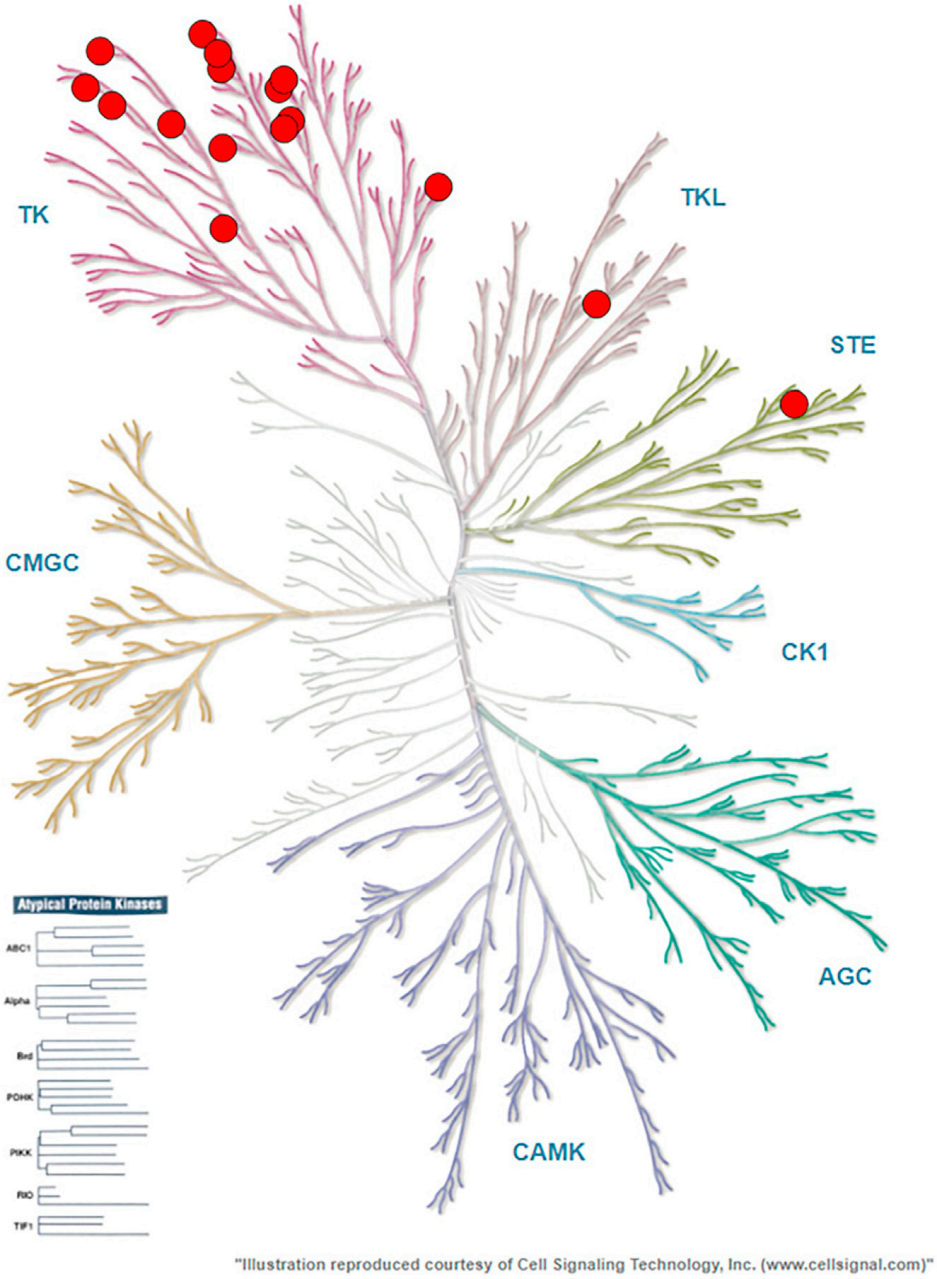
Results of live cell kinase profiling using control compound crizotinib at 1000 nM. Each dot represents a kinase occupied > 50% by the control compound Results are the mean of three independent experiments (n = 3) and summarized in Table 1. Illustrations were reproduced courtesy of Cell Signaling Technologies, Inc.

**Figure 2:**
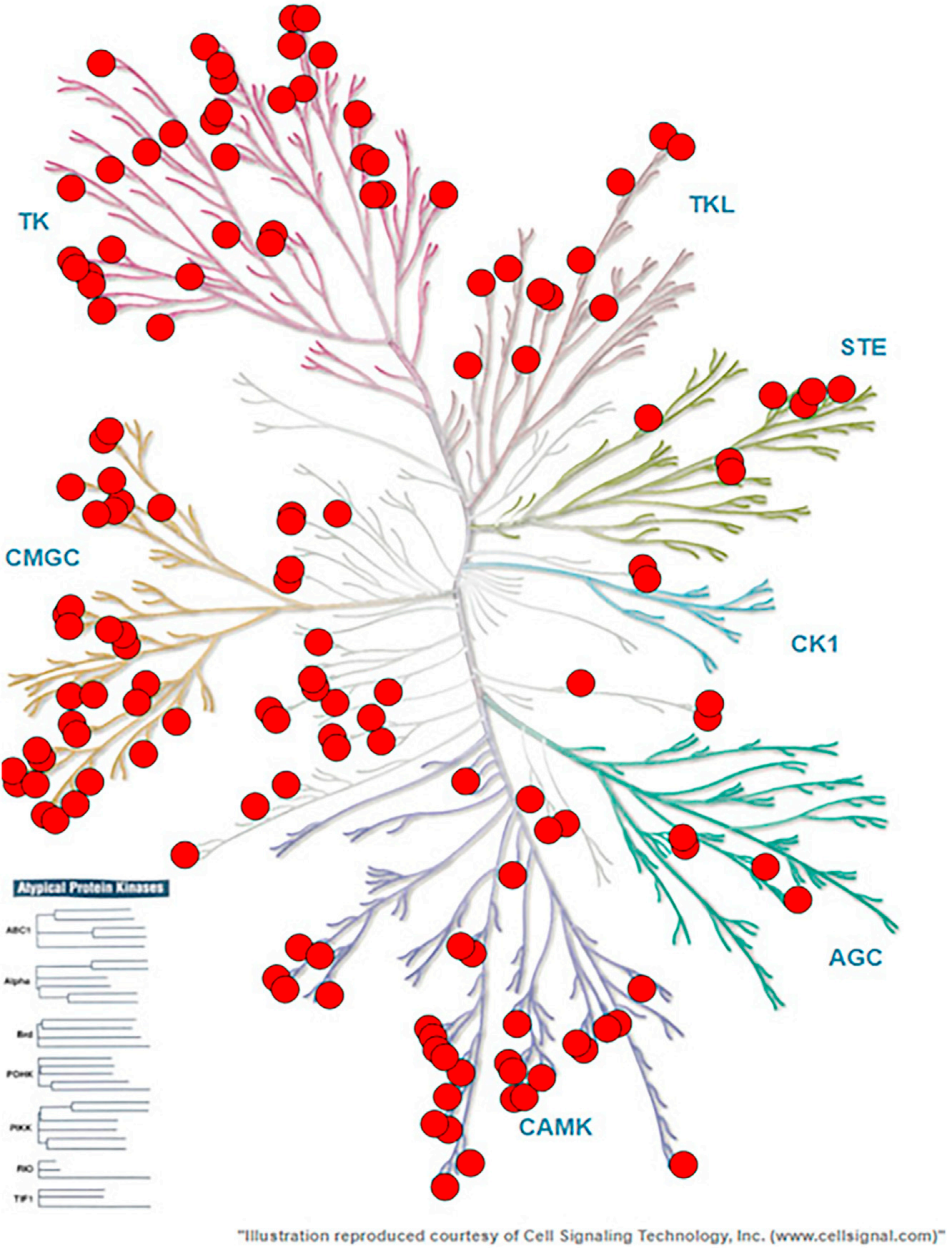
Results of live cell kinase profiling using control compound CC1 at 300 nM. Each dot represents a kinase occupied > 50% by the control compound Results are the mean of three independent experiments (n = 3) and summarized in Table 1. Illustrations were reproduced courtesy of Cell Signaling Technologies, Inc.

Experimental outcome is impacted by a number of variables, which are intrinsic to both the NanoBRET(TM) system, and the test compound of interest. Consult this section of Observed Outcomes when evaluating assay performance across this panel of 192 kinases. A range of variation in BRET signals should be expected, due to variables in BRET efficiency from target to target. Table 1 summarizes the average BRET signal increase observed for each kinase in the presence of tracer K10 alone (absence of test compound). Fractional occupancies are reported for control compounds crizotinib (at 1 μM; Figure 1) and pan-kinase inhibitor CC1 (at 300 nM; Figure 2). For first time users, it may be valuable to compare assay results to this table to ensure accurate experimental setup and BRET detection.

## Limitations

The NanoBRET(TM) method may not be capable of detecting all modes of target engagement. For example, allosteric inhibitors that bind via a mechanism that is non-competitive with the ATP-site tracer may result in undetectable occupancy, thus representing false negatives. It is critical to recognize therefore, that complementary methods may be required to deconvolute some assay results that fail to correlate with those observed using alternate phenotypic or pathway analysis methods.

## Troubleshooting

### Problem 1

Weak expression levels (450 nm RLU < 1000 fold above reagent background) (step 14).

### Potential solution

Weak expression observed in step 14 may be a result of:

Cell status at time of transfection. Ensure that the cells were passaged one day prior to transfection, and that the cell confluency was appropriate (70–90%).
Inaccurate DNA stock solution preparation. Ensure the integrity and concentration of the DNA using standard fluorometric assays. Ensure that the DNA solution did not evaporate during storage. If evaporation occurred, consider adjusting the [DNA] accordingly.
Inaccurate transfection complex preparation. Rely on the transfection control samples to ensure that each experiment results in appropriate transfection levels prior to executing the full kinome profiling experiment. This can save reagent when aberrant transfections occur.

### Problem 2

Noisy BRET S/B, generating coefficient of variations (CV)s > 20% (steps 24 and 25)

### Potential solution

Noisy BRET data observed in step 24 or 25 may be a result of:

Weak expression levels. Ensure that the donor (450 nm channel) RLUs for each kinase are > 1e3 above background (reagent only) control wells
Inconsistent dispensing of tracer. Ensure that liquid handlers are accurately delivering the tracer to each well.

### Problem 3

Negative % occupancy of test compound (step 25).

### Potential solution

Negative fractional occupancy of test compound may be a result of:

Inaccurate dispensing of tracer for DMSO samples (100% BRET, or 0 % fractional occupancy controls). Ensure liquid handling is accurately dispensing the NanoBRET(TM) tracer
Auto-fluorescent or light scattering properties of the test compound. Optical effects may increase the BRET value. This is often determined by using an irrelevant BRET control assay. If the compound has the same effect on an irrelevant BRET assay, this is likely a spurious optical effect.
Although rare, global / nonspecific impacts on kinase activation state may be observed. Non-specific kinase inhibitors may indirectly impact the target of interest, thus increasing the activation state of the kinase. In some cases, increasing the kinase activation state may increase the apparent affinity of the NanoBRET(TM) tracer leading to a non-specific increase in BRET. It may be possible to run specific NanoBRET(TM) kinase assays in digitonin-treated cells to determine if this increase in BRET is due to such non-specific pathway influences as described in earlier studies(Robers et al., 2020; Vasta et al., 2018).

### Problem 4

Unexpectedly low % target occupancy of a test compound (step 25).

### Potential solution

Unexpectedly low % occupancy may be a result of:

Inaccurate dispensing of test compound. Ensure liquid handling is accurately dispensing the compound
Poor compound solubility. Ensure that the compound is soluble as a 10**×** solution.
Discordance between a cell-free and live cell target engagement assay. If comparing NanoBRET(TM) to a cell-free assessment of target occupancy, consider the impact of permeability or [ATP], which may interfere with target engagement. The composite effect of these variables may shift the occupancy results in a live cell vs an acellular system. Follow up experiments in digitonin-treated cells may be warranted to address the impact of [ATP] or permeability as described in earlier studies (Robers et al., 2020; Vasta et al., 2018).

### Problem 5

Unexpectedly high % target occupancy of a test compound (step 25).

### Potential solution

Unexpectedly high % occupancy may be a result of:

Inaccurate dispensing of test compound. Ensure liquid handling is accurately dispensing the compound
Discordance between a cell-free and live cell target engagement assay. If comparing NanoBRET(TM) to a cell-free assessment of target occupancy, consider the impact of target activation state. If the compound preferentially engages an active or inactive kinase state, this may impact intracellular engagement to an unpredictable extent.

## Resource availability

### Lead contact

Further information and requests for resources and reagents should be directed to and will be fulfilled by the lead contact, Matthew Robers (Matt.robers@promega.com).

### Materials availability

The NanoBRET tracer and kinase/Nluc fusion plasmids are each available from Promega. https://www.promega.com/products/cell-signaling/kinase-target-engagement/nanobret-te-intracellular-kinase-assay/?catNum=N2521

## Data Availability

The published article includes all data sets generated or analyzed during this study.
